# Immunomodulatory activity of argentatins A and B isolated from guayule

**DOI:** 10.1371/journal.pone.0304713

**Published:** 2024-05-31

**Authors:** Aniela M. Silva-Nolasco, Miguel A. de la Cruz-Morcillo, M. Mercedes García-Martínez, Amaya Zalacain, Beatriz G. Gálvez, Manuel Carmona

**Affiliations:** 1 Institute for Regional Development (IDR), Universidad de Castilla-La Mancha, Albacete, Spain; 2 Instituto Técnico Agronómico Provincial (ITAP) S.A. Polígono Industrial Campollano, Albacete, Spain; 3 Escuela Técnica Superior de Ingenieros Agrónomos y de Montes y Biotecnología, Universidad de Castilla-La Mancha, Albacete, Spain; 4 Department of Biochemistry and Molecular Biology, Faculty of Pharmacy, Universidad Complutense de Madrid, Madrid, Spain; HNBGU: Hemvati Nandan Bahuguna Garhwal University, INDIA

## Abstract

Argentatins are secondary metabolites synthesized by guayule (*Parthenium argentatum* A. Gray) with numerous potential medical applications. In addition to inhibiting insect growth, they are endowed with several pharmacological properties including antimicrobial and antitumorigenic activity. However, their potential as immunomodulators remains unexplored. The aim of the present study was to investigate whether argentatins can modulate the function of the immune system. Human mesenchymal stem cells were treated with argentatins and the production of several anti- and proinflammatory cytokines was evaluated. The effect of argentatins on the polarization of CD4+ T-lymphocytes and macrophages was also assessed. Results demonstrated that argentatins can modulate the production of proinflammatory cytokines and the polarization of cellular phenotypes, including Th2 lymphocytes and M1 macrophages. These findings suggest that argentatins are promising therapeutic agents in autoimmune or allergic diseases, and open new perspectives for the investigation of argentatins in immune response and in the development of more targeted and effective immunomodulatory therapies.

## Introduction

For many decades, plants have provided us with natural bioactive compounds that benefit our health, often serving as starting points for the synthesis of new medicines. A good example of this is the guayule shrub (*Parthenium argentatum* A. Gray), which is native to the arid landscapes of the Chihuahuan desert in México and the southwestern USA [1]. In addition to its use in the manufacture of hypoallergenic latex, several secondary metabolites isolated from guayule have received increasing attention in recent years due to their unique structural characteristics and biological properties. The most important of these are the argentatins, which are the principal secondary metabolites of guayule resin, accounting for approximately 30% of the total resin obtained from this shrub [2]. Argentatins are cycloartane-type triterpene compounds with diverse pharmacological properties, including antimicrobial and anticarcinogenic activity *in vitro*. For example, used at a concentration of 238 μM, argentatin A was found to inhibit the growth of the pathogenic yeasts *Candida albicans* and *Torulopsis glabrata* better than chloramphenicol, which was used as a control, and also showed inhibitory activity against *Klebsiella pneumoniae* and *Pseudomonas aeruginosa* when compared with fluconazole [3]. Regarding their anticarcinogenic properties, both argentatin A and B are effective in inhibiting up to 100% of the growth (at 100 μM) of various cancer cell lines [4], thus showing very promising results in terms of activity.

Another interesting property of the argentatins is their potential anti-inflammatory activity. Although studies are scarce, argentatins have been shown to reduce inflammation in *in vivo* models of edema induced by tetradecanoylphorbol acetate; argentatin B used at 15 μM inhibited COX-2 activity by 77%, which was superior to indomethacin used as a reference [5]. In the same study, the effect of argentatins A and B on nitric oxide (NO) production was evaluated in mouse peritoneal macrophages stimulated with lipopolysaccharide to induce the release of tumor necrosis factor alpha (TNF-α) and NO. Results showed that both compounds at 31 and 100 μM significantly inhibited the release of nitrites, which are the precursors of NO. Moreover, in metabolic assays, argentatin A and B used at 50 μM reduced the cell viability of resting macrophages by 63% and 53%, respectively, suggesting that the reduction in NO is directly related to their influence on cell viability [5].

An area of growing interest in drug development is immunomodulation, which involves reshaping the host response through therapeutic strategies that enhance or suppress the immune system response as needed. A classical example of immunomodulation is in the treatment of autoimmune diseases such as rheumatoid arthritis, systemic lupus erythematosus and multiple sclerosis, which is applied to suppress the immune response and reduce inflammation and associated tissue damage [6]. Immunomodulatory drugs are also used to prevent organ and tissue rejection after transplantation [7]. A more recent example of immunomodulation is the use of checkpoint inhibitor-based immunotherapies to block the signals that inhibit the immune response against cancer cells [8]. Also, immunodulatory drugs are used in the context of inflammatory and allergic diseases involving an exaggerated immune response, such as asthma and atopic dermatitis [9, 10]. Finally, there has been an interest in targeting immunomodulation in neurodegenerative disorders such as Alzheimer’s and Parkinson’s disease to treat brain inflammation [11].

Inflammation is a biological protective response to injury, infection, irritation or cellular damage that eliminates infectious agents, removes dead and damaged cells and facilitates the tissue repair process. Its mechanism is complex and involves many different cells of the immune system. The inflammatory response consists of the activation of resident immune cells such as mast cells and macrophages, which recruit leukocytes (neutrophils and monocytes) to the site of injury or infection to repair tissue damage and kill pathogens [12]. It is important to note that although the response to acute infection is usually rapid and transient, inflammation can become chronic. This has been linked to several diseases, including autoimmune, cardiovascular and metabolic disorders, as well as certain types of cancer [13].

The effect of argentatins on the development of cellular immune responses and cytokine production has not yet been evaluated. Accordingly, the present study was designed to investigate the potential of argentatin A and B to shape the immunomodulatory properties of mesenchymal stem cells (MSCs), and to assess their direct effects on immune cells to better understand their potential ability to modulate inflammation.

## Material and methods

### Chemical and reagents

Dulbecco’s modified Eagle’s medium (DMEM)-high glucose, Roswell Park Memorial Institute (RPMI) 1640 medium, fetal bovine serum (FBS), phosphate buffered saline (PBS), dimethyl sulfoxide (DMSO), 3-(4,5-dimethylthiazol-2-yl)-2,5-diphenyltetrazolium bromide (MTT), lipopolysaccharide (LPS), interferon gamma (IFN-γ), macrophage colony-stimulating factor (M-CSF) and human IgG were purchased from Sigma-Aldrich (St Louis, MO, USA). Antibodies for lymphocyte differentiation were purchased from Abcam (Cambridge, UK) (IL-4, IL-10, rIL-2, rIL-12, IL-23p40, IL-4, rIL4, IL-12p40, anti-CD3 and anti-CD28). Penicillin and streptomycin were from Gibco (Gaithersburg, MD, USA). MSCs were purchased from Lonza (Basel, Switzerland) and THP-1 monocytes and CD4+ T-lymphocytes were from the ATC (Manassas, VA, USA). Argentatins A and B were isolated from guayule resin as described [14] with a purity of 88% and 87%, respectively, and were dissolved in DMSO at 20 mM as stock solutions, which were stored at -80°C.

### Cell culture and growth conditions

Human MSCs (PT-2501) were plated (1 × 10^6^) in a 100-mm plate and cultured for 48 hours in complete DMEM/10% FBS, as described [15]. When the plate was near confluent the culture supernatant (conditioned medium) was collected and centrifuged at 1,200 × g at room temperature for 10 minutes. The upper aqueous solution was aliquoted in 1.5-ml microtubes and kept at -80°C until use.

THP-1 human leukemia monocytes (TIB-202) were first cultured in RPMI 1640 supplemented with 5% FBS and 10 ng/ml M-CSF at 37°C and 5% CO_2_ for 72 hours, and then in the same medium without FBS for a further 48 hours. To induce differentiation to an M1 (proinflammatory) phenotype, the medium was supplemented with 100 ng/ml LPS and 25 U/ml IFN-γ. To induce differentiation to an M2 (anti-inflammatory) phenotype, the medium was supplemented with 20 ng/ml IL-4 and 20 ng/ml IL-10.

CD4+ T-lymphocyte cells (PCS-800-016) were grown in complete RPMI medium and their differentiation into Th1 or Th2 subpopulations was performed as described [16, 17]. Briefly, CD4+ T-cells were cultured in complete RPMI medium and activated with 1 μg/ml anti-CD3, 2 μg/ml anti-CD28 and 100 U/ml rIL-2. For differentiation into Th1 cells (Th1- differentiation condition) 2.5 ng/ml of rIL-12/IL-23p40 and 50 ng/ml of anti-IL-4 were added. For differentiation into Th2 cells (Th2- differentiation condition) 12.5 ng/ml of rIL-4 and 1 μg/ml of anti-IL-12p40/70 were added. Differentiation was evaluated after 5 days of culture.

There were no reports that cell line used in the assays was misidentified or cross-contaminated [18].

### Proliferation assay

The viability and proliferation of MSCs upon treatment with argentatins was measured with an MTT reduction assay and spectrophotometry [19]. MSCs were seeded into 96-well tissue culture plates at 1 × 10^5^ cells per well in 0.2 ml of DMEM-high glucose supplemented with 10% FBS and 1% penicillin/streptomycin and grown at 37°C in a humidified 5% CO_2_ incubator for 24 hours. Cells were then treated for 24 or 48 hours in the presence of different amounts of argentatins (20, 50 and 100 μM). DMSO alone was used in control conditions. Relative cytotoxicity was expressed as a percentage of [ODsample − ODblank]/[ODcontrol − ODblank] × 100. Each experiment was performed in triplicate.

### Flow cytometry analysis

Monocytes and differentiated macrophages were harvested by gentle scratching with a cell scraper (Sarstedt, Newton, NC, USA) and T-lymphocytes were collected after centrifugation. In both cases, cells were resuspended in serum-free RPMI (2 × 10^6^ cells/ml) and incubated for 30 minutes on ice with 150 μg/ml of Human IgG to block Fc receptors. Cells were analyzed on a FACsCanto II flow cytometer (Becton Dickinson, Sunnyvale, CA, USA). Sample acquisition was performed on FACSVerse with FACSuite v.1.0.6.5230 (BD Biosciences) and data analysis was done using FlowJo software (all from Becton Dickinson).

Macrophages were stained with 20 μg/ml of the corresponding primary antibody on ice for 30 minutes. The mixtures of monoclonal antibodies used for detection of the different populations are shown in S1 Table. After 5 days of differentiation in the presence or absence of argentatins, T-helper CD4^+^ lymphocytes were gated by positive surface staining for CD4 and were expressed as a percentage of gated lymphocytes; Th1 cells (CD4+IFN-c+) and Th2 cells (CD4+IL-4+) were expressed as a percentage of CD4+ cells. Macrophage analysis and data acquisition was performed as described for monocytes [20].

### Cytokine assay

The concentration of several cytokines was determined using commercial ELISA detection kits (Merck Millipore, Darmstadt, Germany) with a fluorescence plate reader (BioTek Instruments, Winooski, VT, USA). The level of cytokines (IL-2, IL-4, IL-6, IL-10, TNF, INF-γ, IL17-A) in supernatants was measured after collection of conditioned medium of cultured cells, as previously reported [21] in the presence or absence of argentatins.

### Statistical analysis

The results are represented as the mean and standard error of the mean (SEM); the Shapiro-Wilk test was applied to check whether the distribution was normal. A parametric analysis of variance (ANOVA) with Dunnet’s post test was used to compare each study group with respect to the control group (for cell proliferation and cytokine release assays), and a Tukey’s post test for cell differentiation assays, where a positive and a negative control were compared; a value of p<0.05* was considered statistically significant and a value of <0.0005*** highly significant. Statistical analysis was performed with GraphPad Prism software version 9.5.1, (GraphPad Prism, Software San Diego, CA, USA).

## Results and discussion

### Cell viability assay

To evaluate the potential toxicity of the argentatin A and B compounds isolated from guayule resin, we performed a cytotoxicity assay based on the reduction of a yellow tetrazolium salt (3-(4,5-dimethylthiazol-2-yl)-2,5-diphenyltetrazolium bromide, MTT). Because the argentatins are antiproliferative in several *in vitro* tumor models, including HCT15, HCT116, SW620 and RKO cells [22], it was important to assess whether the compounds were cytotoxic in the MSC model. Accordingly, MSCs were treated with increasing doses of argentatin A and B (20, 50 and 100 μM) based on the literature, for 24 and 48 hours.

**Fig 1:**
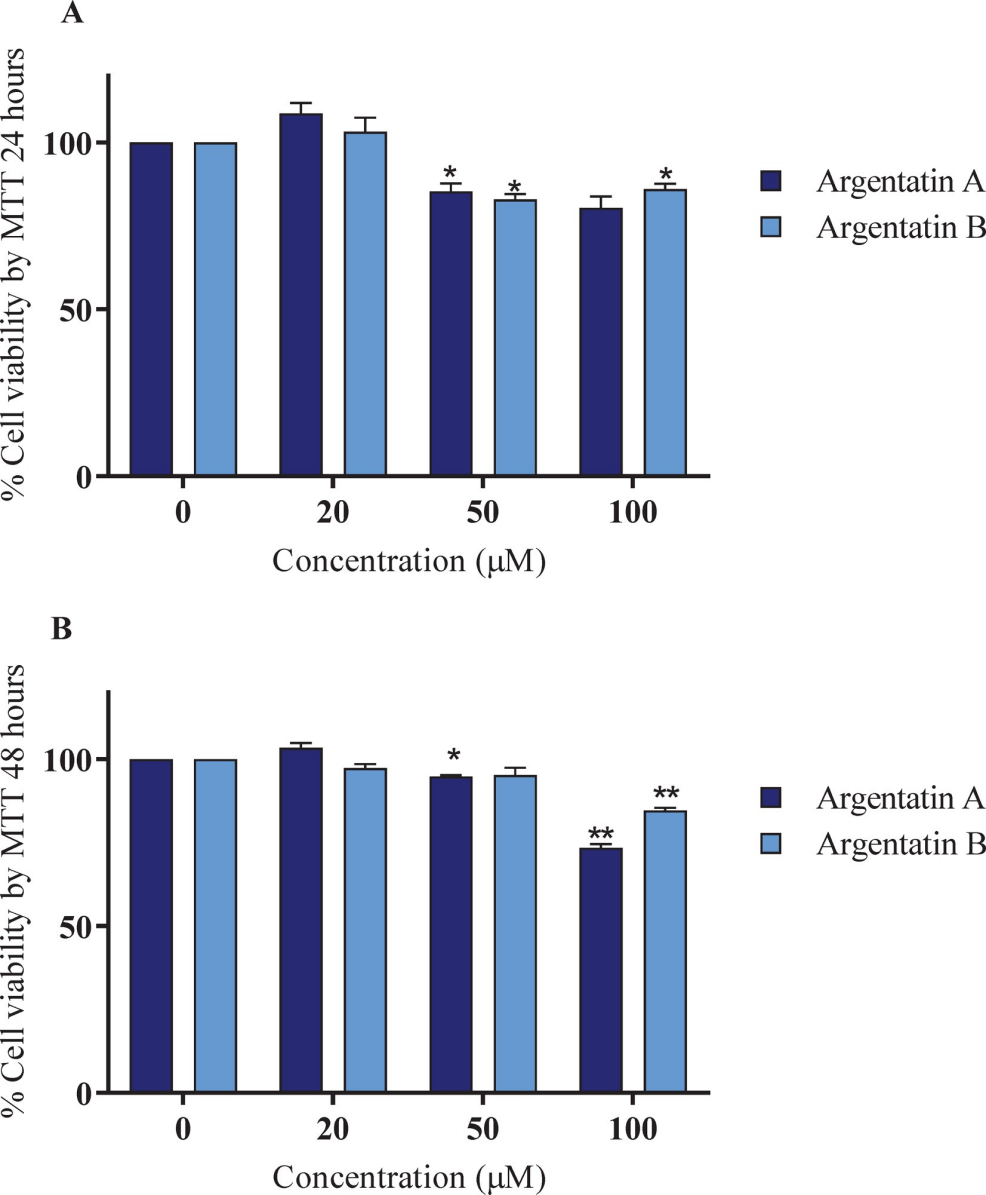
Viability of mesenchymal stem cells (MSCs) in response to argentatin A and B. (A) Viability at 24 h, (B) Viability at 48 h. Cell viability was determined by MTT reduction and was compared to the corresponding untreated control (DMSO), which was normalized to 100% cell viability. Error bars represent the standard error of the mean (SEM) of three independent experiments. Two-way ANOVA with Dunnet´s post test was used to determine differences between argentatin A and B treatments compared with control (*p<0.05, **p<0.005).

No toxicity was detected for MSCs at 20 μM for both argentatins compared with control (DMSO) after 24 hours (Fig 1A). Nevertheless, argentatin A and B were cytotoxic at the higher concentration of 50 μM (p = 0.0481 and p = 0.0160, respectively). At the highest dose of 100 μM, the cytotoxicity against MSCs was very similar (Fig 1A).

These data indicate that concentrations exceeding 50 μM should be avoided for MSCs. After 48 hours of treatment, 50 μM argentatin A exerted a slight but significant cytotoxicity compared with the control (p = 0.0113), but no significant toxicity was observed for argentatin B (Fig 1B). The dose of 100 μM for argentatin A and B was discarded for future studies because of its high toxicity (p<0.0031 and p<0.0251, respectively), with argentatin A being slightly more cytotoxic than argentatin B (Fig 1B). Of note, neither compound led to a cell viability lower than 50% at the highest concentration and treatment duration, contrary to what has been observed in tumor cell lines with a 50% inhibitory concentration at doses between 20 and 30 μM [4, 23].

Some anticancer compounds tend to have certain selectivity only for cancer cells. This may be due to several reasons, such as the difference in proliferation rate, as they tend to divide and grow faster than normal or healthy cells. Or the genetic mutations that cancer cells tend to have and differentiate them from normal cells, so, some anticancer compounds are designed precisely for this, to target specific processes related to cell division, affecting cells that divide more frequently such as cancer cells. Or they are designed to target mutated or overexpressed proteins, without affecting normal cells as much. Such is the case of the selectivity of curcumin, where its potent action against cancer cells has been demonstrated and its immunomodulatory effect evidenced by good bone marrow activity after administration with no apparent toxicity in the normal cells and even in a murine model, justified by normal behavior and the absence of death or health deterioration [24–26]. This is potentially significant as it could inform new strategies targeted at treating autoimmune and inflammatory diseases with MSCs, which are involved in key functions such as tissue repair and regeneration due to their capacity to differentiate into different cell types and to release immunomodulatory and anti-inflammatory factors [27].

### Cytokine expression

**Fig 2:**
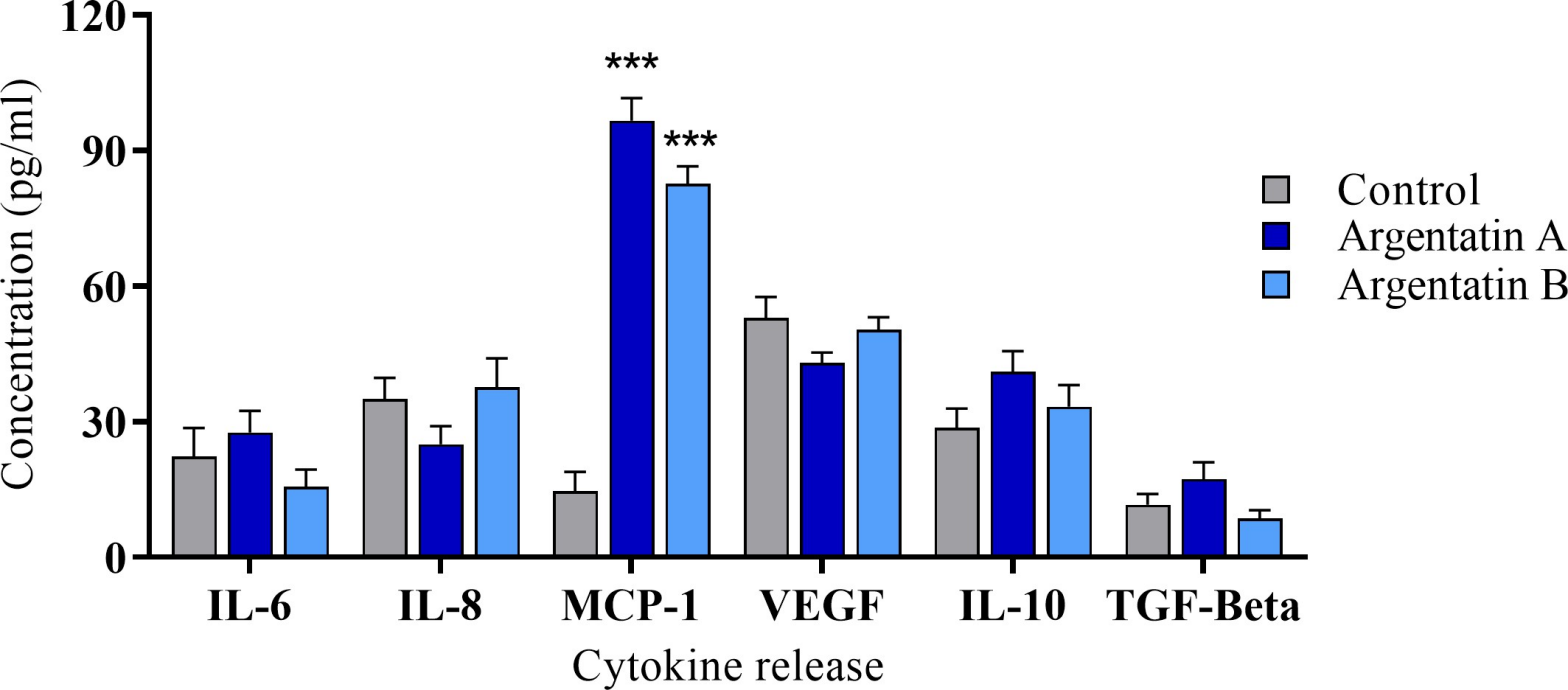
Production of anti-inflammatory and proinflammatory mediators in argentatin-stimulated MSCs. Cytokine production by MSCs was evaluated in the absence or presence of stimulation for 24 hours at 50 μM. Error bars represent the standard error of the mean (SEM) of three individual experiments. Two-way ANOVA with Dunnet´s post test was used to determine differences between argentatin A and B treatments compared with control (***p<0.0005).

We next questioned whether argentatin treatment of MSCs promoted cytokine production. MSCs were treated with 50 μM argentatin A and B for 24 hours and the levels of six pro- or anti-inflammatory cytokines were quantified from conditioned medium by ELISA. No significant changes were observed for the production of the anti-inflammatory cytokines IL-10 and TFG-β or for the proinflammatory cytokines IL-6, IL-8 and VEGF (Fig 2).

However, results revealed a marked increase in the production of the proinflammatory cytokine MCP-1 after treatment with either argentatin (Fig 2). MCP-1 (also called chemokine [CC-motif] ligand 2) has a crucial role in immunomodulation by participating in the attraction and regulation of monocytes and macrophages, which in turn differentiate into M1 or M2 cells [28]. It thus contributes to both acute and chronic inflammation.

### Cellular differentiation

**Fig 3:**
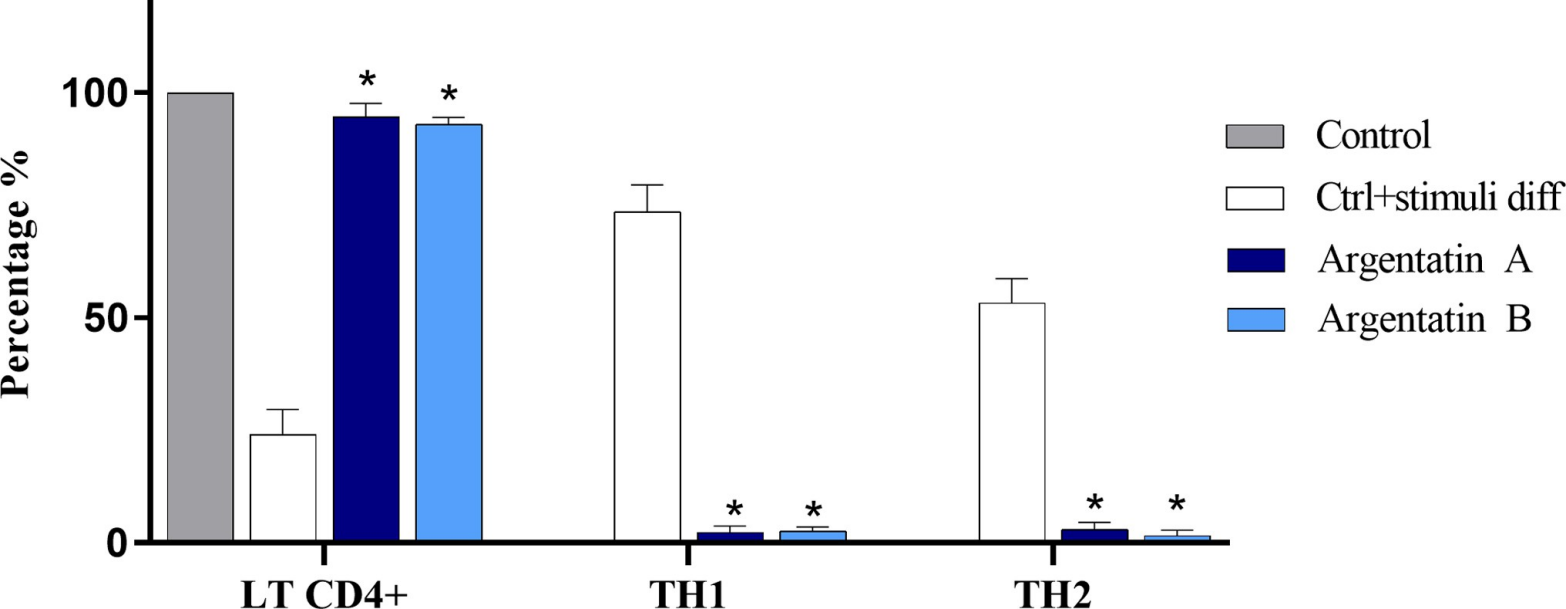
Percentage of LT CD4+ differentiated to Th1 and Th2 subtypes by argentatins. The treatment with both argentatins was at 50 μM and the differentiation process was evaluated by cytometry after 5 days of incubation. Error bars represent the standard error of the mean (SEM) of three individual experiment and the data can be found in S2 Table. Two-way ANOVA with Tukey´s post test was used to determine differences between argentatin A and B treatments compared with positive control (*p<0.05). No statistically significant differences were found with respect to the negative control.

CD4+ T lymphocytes play a critical role in regulating the immune response and can differentiate into specific subtypes based on the presence of cytokines and other signals in the microenvironment, in addition to the levels of antigens, the type of antigen-presenting cell and costimulatory molecule [29]. Given our finding of a marked increase in MCP-1 production following argentatin treatment, we next evaluated whether argentatin A and B could influence the differentiation of CD4+ T lymphocytes to Th1 and Th2 cells, which are the most common subtypes. As negative control for this study we used CD4+ T cells without differentiation factors, and as a positive control we used CD4+ T cells with full differentiation factors (see materials and methods). In the presence of argentatin A (50 μM for 5 days), differentiation of the lymphocyte population to the Th1 phenotype, which is known for facilitate humoral responses, was 2% with respect to the negative control, and differentiation to the Th2 phenotype, which mediates delayed type hypersensitivity responses and inflammatory reactions, was about 3% (Fig 3). Both cell types normally function in a balanced manner, however, exposure to pathogens may cause one subset to predominate. The results for argentatin B treatment were very similar with values of 3% and 2% for the Th1 and Th2 phenotypes, respectively.

Chronic inflammation is a consequence of the predominance of Th1 response, whereas allergy and hypergammaglobulinemic states are caused by the predominance of Th2 responses [16]. While the argentatins had only a modest effect on cell differentiation, there was a relative equivalence in the lack of polarization between the two lymphocyte phenotypes, meaning that an unbalanced immune response such as in the case of autoimmune or allergic diseases would not occur. However, it is important to note that the manipulation of differentiation will depend on the context and the state of activation of the immune system, as this process is essential in many situations such as fighting infections.

To further evaluate the immunomodulating role of argentatins, their effect on the phenotypic differentiation of macrophages was studied. The monocytic cell line THP-1 was chosen to examine macrophage differentiation because of the similarities it shares with primary monocytes, such as morphology and physiology, minimizing cell phenotype variation arising from monocyte-to-macrophage differentiation by using a single source of cells [30]. Similar to lymphocytes, THP-1 macrophage polarization to the principal phenotypes M1 and M2 are processes controlled by the microenvironment and associated signals.

**Fig 4:**
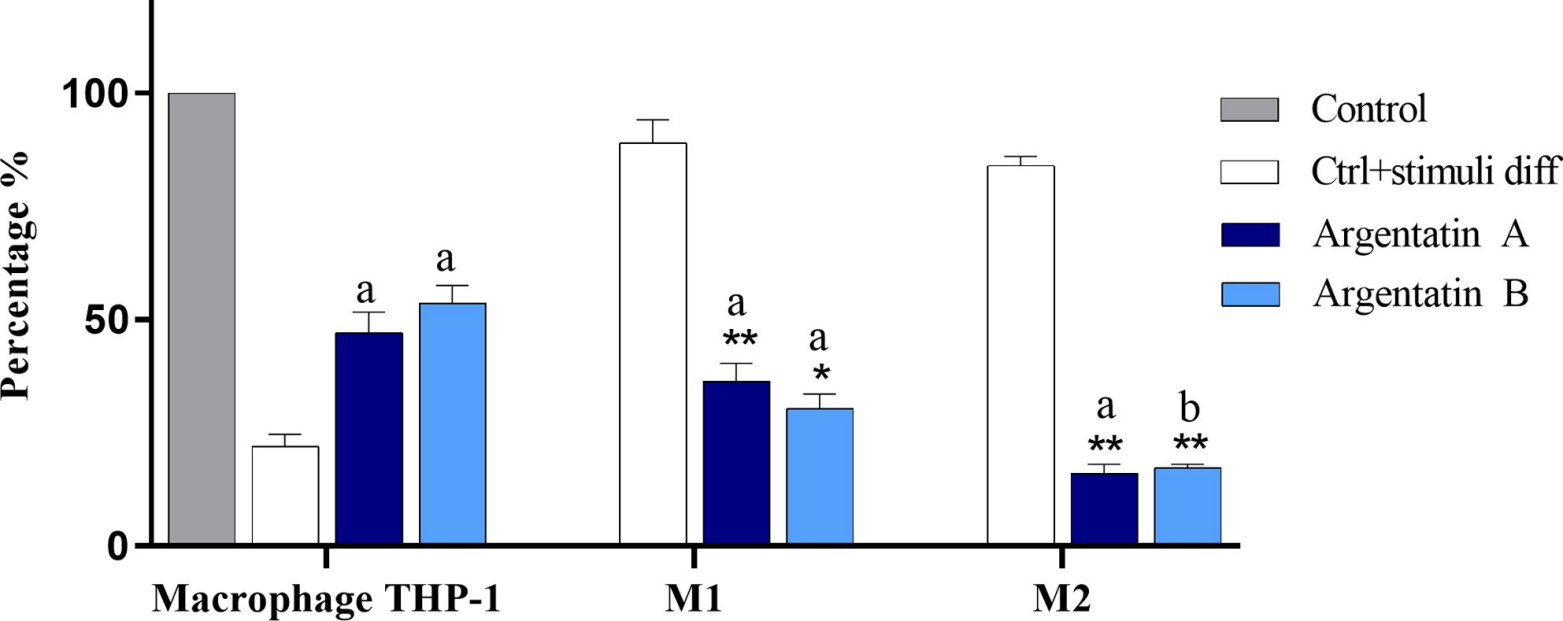
Percentage of THP-1 macrophages differentiated to M1 and M2 subtypes by argentatins. The treatment with both argentatins was at 50 μM and the differentiation process was evaluated by cytometry after 3 days of incubation. Error bars represent the standard error of the mean (SEM) of three individual experiments and the data can be found in S2 Table. Two-way ANOVA with Tukey´s post test was used to determine differences between argentatin A and B treatments compared with controls. The letters refer to the significant difference compared to the negative control and the asterisk compared to the positive control (*p<0.05, **P<0.005, (a) P<0.05, (b) P<0.005).

Treatment of THP-1 cells with argentatin A (50 μM for three days) stimulated M1-type macrophage differentiation almost 40-fold greater than that observed in the negative control, whereas M2-type differentiation was about 16-fold higher compared to the negative control (Fig 4). Similarly, the equivalent treatment of THP-1 cells with argentatin B stimulated M1-type macrophage differentiation 30-fold higher than the negative control, a lower percentage than argentatin A, whereas M2-type differentiation was 17-fold higher with respect to the negative control (Fig 4). The function of M1-type macrophages is related to inhibiting cell proliferation and causing tissue damage, whereas M2-type macrophages promote cell proliferation and tissue repair [31].

Most studies performed in the search for natural immunomodulatory compounds involve strategies to resolve inflammation by increasing M2 levels and/or decreasing M1 levels, rather than directly promoting differentiation to M1. Compounds such as polysaccharide isolated from the goji plant (*Lycium barbadum*) have been tested both *in vitro* and *in vivo* at a concentration of 10 mg/kg and 100 μg/ml, respectively, in microglial cells, and have been found to induce polarization to M1 macrophages, and a similar result have been noted for *Platycodon grandifloras* polysaccharide in the RAW 264.7 macrophage line at concentrations of 0.01 to 10 μM [32, 33].

In the context of cancer, the propensity for macrophages to polarize to the M1 phenotype may have benefits by secreting proinflammatory cytokines such as TNF-α and IFN-γ, thereby helping to activate an appropriate immune response against cancer cells. This is the case for curcumin (8 μM) in the presence of LPS, which was tested in the A549 lung cell line, and was found to promote the secretion of IL-1β and TNF-α by reprogramming M2 macrophages into tumor-killing M1 macrophages [34]. The same effect was observed with astragalus polysaccharide (16 mg/ml) in the NSCLC cell line H441 co-cultured with monocyte-derived macrophages, and with β-glucan (800 μM) in mouse models implanted with Lewis lung carcinoma cells (LLC) or EO77 cells, which significantly reduced IL-10 levels while increasing the mRNA levels of iNOS, IL-12, TNF-α, IL-1β and IL-6 [35, 36]. Another example is the polysaccharide portion of ginseng berry (GBPP), which at 100 μg was found to promote the production of cytokines such as TNF-α and IL1-12, thus inducing M1 polarization in mouse lung cancer models [37].

Although Th1 and Th2 cells can secrete cytokines in certain contexts that influence the polarization of M1 and M2 macrophages, respectively, they are not the only factor that determines polarization, as shown in this study, where the predominant phenotype was M1 after treatment with both argentatins irrespective of the low differentiation capacity for Th1 cells [38].

Beyond the secretion of inflammatory mediators, M1 cells can directly phagocytose and destroy cancer cells through the production of reactive oxygen and nitrogen species, as well as by promoting adaptive immunity by stimulating the response of cytotoxic T cells (CD8+). This favors the presentation of tumor antigens, which would lead to the enhancement of antitumor immunity [39, 40].

Nonetheless, the balance between M1 and M2 phenotypes in the tumor context may change during cancer progression. Therefore, the investigation of targeted therapies to modulate polarization in macrophages is a promising research approach.

## Conclusions

Here we examined the effects of argentatins A and B on MSCs and their potential to regulate the immune response. We found that at doses of 20–50 μM both compounds were not toxic to MSC cells, making them safe for future use. Also, argentatin A promoted the proliferation of MSCs, which could be beneficial for tissue regeneration and immune system modulation. This is supported by the finding that both argentatin A and B, increased the release of MCP-1 and also favored the differentiation of immune cells (CD4+ T lymphocytes and THP-1 macrophages). To the best of our knowledge, this is the first demonstration of an immunomodulatory function of argentatins, but further research is needed to fully understand their effects on the regulation of immune response and inflammation, which might open up new therapeutic avenues for the development of treatments for several diseases with an inflammatory component.

## Supporting information

S1 Table Monoclonal antibody mix used for the assessment of macrophage populations by flow cytometry. (PDF)

S2 Table Percentages of differentiated cells by argentatins. LT CD4+ differentiated to Th1 and Th2 cell subtypes, and THP-1 macrophages differentiated to M1 and M2 cell subtypes respectively.
(PDF)

S1 Raw data (XLSX)
