# Supplementary material for: Immunomodulatory activity of argentatins A and B isolated from guayule
Source: PLoS One. 2024 May 31;19(5):e0304713. doi: 10.1371/journal.pone.0304713 (PMC11142701; doi:10.1371/journal.pone.0304713)
Supplement: S1 Table — (PDF) [file pone.0304713.s001.pdf]

Specificity	mAb clone	Isotype	Fluorochrome	Volume	Manufacturer	Cat. No
CD3	UCHT1	IgG1, k	A700	1 µl	BioLegend	300424
CD14	HCD14	IgG1, k	APC/Cy7	2.5 µl	BioLegend	325620
CD19	HIB19	IgG1, k	A700	1 µl	BioLegend	302226
CD45	HI30	IgG1, k	Pacific Blue	2.5 µl	BioLegend	304029
CD163	RM3/1	IgG1, k	APC	2.5 µl	BioLegend	326509
CD204	7C9C20	IgG2a, k	PE/Cy7	2.5 µl	BioLegend	371907
CD206	15-2	IgG1, k	A488	2.5 µl	BioLegend	321113
CD209	9E9A8	IgG2a, k	PE	2.5 µl	BioLegend	330105
HLA-DR	L243	IgG2a, k	BV605	2.5 µl	BioLegend	307640
