# Supplementary material for: Immunomodulatory activity of argentatins A and B isolated from guayule
Source: PLoS One. 2024 May 31;19(5):e0304713. doi: 10.1371/journal.pone.0304713 (PMC11142701; doi:10.1371/journal.pone.0304713)
Supplement: S2 Table — LT CD4+ differentiated to Th1 and Th2 cell subtypes, and THP-1 macrophages differentiated to M1 and M2 cell subtypes respectively. (PDF) [file pone.0304713.s002.pdf]

	LT CD4+			TH1			TH2		
**Control**	100	100	100	0	0	0	0	0	0
**Ctrl+stimuli diff**	35	21	16	78	62	81	64	46	50
**Argentatin A**	96	89	99	2	5	0	4	0	5
**Argentatin B**	94	95	90	4	3	1	1	4	0

	Macrophage THP-1			M1			M2		
**Control**	100	100	100	0	0	0	0	0	0
**Ctrl+stimuli diff**	21	18	27	92	96	79	85	80	87
**Argentatin A**	47	55	39	43	37	29	15	20	13
**Argentatin B**	52	61	48	36	30	25	18	18	16
